# A Study of the Buffer Capacity of Polyelectrolyte Microcapsules Depending on Their Ionic Environment and Incubation Temperature

**DOI:** 10.3390/ijms23126608

**Published:** 2022-06-14

**Authors:** Alexey V. Dubrovskii, Aleksandr L. Kim, Egor V. Musin, Sergey A. Tikhonenko

**Affiliations:** Institute of Theoretical and Experimental Biophysics Russian Academy of Science, Institutskaya St., 3, 142290 Puschino, Moscow Region, Russia; dav198@mail.ru (A.V.D.); kimerzent@gmail.com (A.L.K.); eglork@gmail.com (E.V.M.)

**Keywords:** polyelectrolyte microcapsules, microcapsules, buffer capacity, polyelectrolyte

## Abstract

Polyelectrolyte microcapsules (PMCs) are used in the development of new forms of drugs, coatings and diagnostic systems. Their buffer capacity, depending on the conditions of the medium, has not been practically studied, although it can affect the structure of both the capsule itself and the encapsulated agents. In this connection, we studied the buffer capacity of polyelectrolyte microcapsules of the composition (polystyrene sulfonate/polyallylamine)_3_ ((PSS/PAH)_3_) depending on the concentration and the type of salt in solution, as well as the microcapsule incubation temperature. It was found that the buffer capacity of microcapsules in the presence of mono- and di-valent salts of the same ionic strength did not differ practically. Increasing the NaCl concentration to 1 M led to an increase of buffer capacity of PMCs at pH ≥ 5, and an increase in NaCl concentration above 1 M did not change buffer capacity. The study of the buffer capacity of pre-heated PMCs showed that buffer capacity decreased with increasing incubation temperature, which was possibly due to the compaction of the PMCs and an increase in the number of compensated PAH sites. The addition of 1 M sodium chloride to heated PMCs presumably reversed the process described above, since an increase in the ionic strength of the solution led to an increase of the buffer capacity of the PMCs. The effects described above confirm the hypothesis put forward that the buffer properties of microcapsules are determined by uncompensated PAH regions in their composition.

## 1. Introduction

Polyelectrolyte microcapsules are supramolecular systems created by the layer-by-layer method [1]. It is applied to develop a target delivery system, self-healing materials, imaging, diagnostic and theranostic systems [2,3,4,5,6,7,8,9,10,11]. The protonation level of such systems may affect both the structure of the system and its encapsulated substances. It is necessary to consider this condition when producing supramolecular systems, so as to decrease the negative effect on encapsulated substances.

Dubrovskii et al. have already demonstrated differences in the behavior of the buffer capacity of polyelectrolyte microcapsules depending on the composition of the PMC shell, the presence of an encapsulated protein, and 0.5 M sodium chloride [12]. We assume that the buffer capacity of PMCs can also be affected by the magnitude of the ionic strength and the temperature of the solution, because there are several works describing their influence on the buffer capacity of free polyelectrolytes and their complexes [13,14,15].

A change in the physicochemical properties of polyelectrolytes under the action of the ionic strength of the solution may be one of the possible reasons for the change in the buffer capacity of polyelectrolyte microcapsules [13,16,17]. It was shown that the addition of 10–150 mM NaCl to a polyethyleneimine solution led to a decrease in the electrostatic repulsion between the charged groups of the polyelectrolyte monomers [16,17]. With a decrease in this repulsion, hydrophobic and hydrogen intramolecular interactions weaken, which increases solubility and hydrodynamic radius [16]. As a result, the effect described above leads to an increase in the amount of solubilized amino groups of the polyelectrolyte capable of protonation/deprotonation.

Another reason for the change in the buffer capacity of polyelectrolyte microcapsules under the action of ionic force may be a change in the solubility of the polyelectrolyte complex (PC) [14,18,19]. It was shown that at concentration of NaCl < 0.3 M the number of compensated regions of the poly(L-Lysine)/poly(L-Glutamic acid) polyelectrolyte complex decreases, while maintaining its integrity [18]. At the same time, the polyelectrolyte complex had physicochemical properties (effective Kuhn length, epsilon and beta correlation lengths) closer to individual polyelectrolyte solutions and, as a result, had higher solubility than the complex in water.

The temperature of the solution can also affect the change in the buffer capacity of PMCs [15,20,21]. It was shown that an increase in incubation temperature led to a decrease in the contribution of water to the free volume of the PC’s polyelectrolytes, an increase in the ionic interaction between PE chains, and a decrease in the slip of PE chains relative to each other [21]. As a result of the effects described above, the solubility of the polyelectrolyte complex was significantly reduced and the number of groups capable of protonation/deprotonation decreased.

In addition, a number of works show changes in the PMC morphology, their cut thickness, and the migration of their polyelectrolyte layers, depending not only on the composition of the PMC shell, the presence of an encapsulated protein, or the presence of sodium chloride, but also on the ionic strength of the solution and the temperature of the medium [22,23,24,25,26,27,28,29,30,31,32,33]. This may also indicate a possible dependence of the buffer capacity of polyelectrolyte microcapsules on these conditions. Thus, the purpose of our work was to study the buffer capacity of various types of polyelectrolyte microcapsules in pH range 4 to 9, depending on ionic strength and temperature of the medium.

## 2. Results and Discussion

In our previous work, we revealed the presence of a buffer capacity (BC) in PMCs consisting of PAH and PSS [12]. It was also found that BC microcapsules increase in the presence of salt in the pH range 5.5 to 9, and it was hypothesized that BC microcapsules correspond to unpaired sections of PAH in their composition. To confirm this hypothesis, we carried out a series of experiments to study the influence of the ionic strength of the solution and the type of salts on buffer capacity of PMCs.

### 2.1. Study of the Buffer Capacity of Microcapsules Depending on the Type of Salt

**Figure 1:**
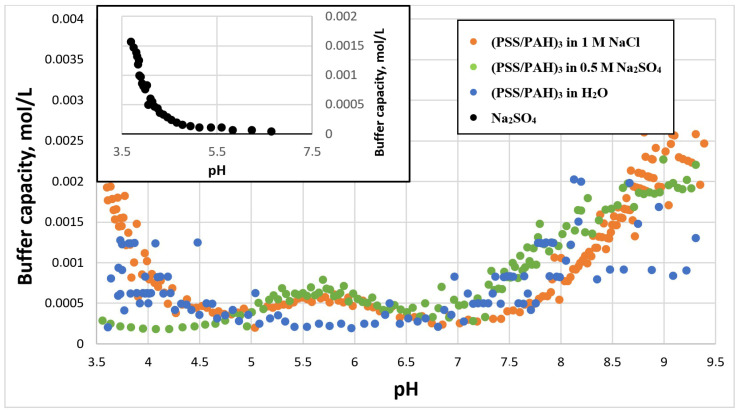
Buffer capacity of PMC composition (PSS/PAH)_3_ at different pH in water, 1 M NaCl and 0.5 M Na_2_SO_4_.

We studied BC of PMCs in the presence of 1 M sodium chloride and 0.5 M sodium sulfate, which have the same ionic strengths at these concentrations. The results of this study are presented in Figure 1.

As can be seen from Figure 1, in the pH range 5 to 9 the BC of PMCs practically did not differ depending on the type of salt. However, at pH less than 4.7, BC of PMCs in sodium sulfate rose sharply, while BC did not grow in sodium chloride. This effect was directly related to the change in the buffer capacity of the solution of the corresponding salts and was not related to the PMCs (Figure 1 insertion).

### 2.2. Study of the Buffer Capacity of PMC in Different Salt Concentrations

**Figure 2:**
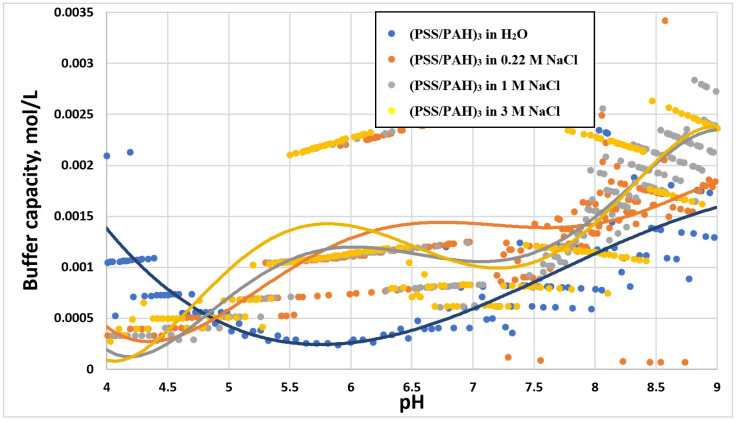
Buffer capacity of PMC composition (PSS/PAH)_3_ at different pH in water, 0.22 M, 1 M and 3 M NaCl solution.

The next stage of the study was to examine the BC of PMCs in the presence of salts to assess dependence on salt concentration. Figure 2 shows the dependence curves of the buffer capacity of the PMC composition (PSS/PAH)_3_ on pH in the presence of various concentrations of sodium chloride.

As can be seen from the figure, the presence of NaCl in the solution led to an increase of BC of the PMCs at pH ≥ 5. At the same time, BC increased with an increase in NaCl concentration to 1 M, which might have been due to a decrease in the number of compensated regions of PMC polyelectrolyte complexes with an increase in the ionic strength of the solution. With an increase in salt concentration from 1 to 3 M, the BC did not change, which was presumably due to the absence of a change in the amount of unbound PAH with an increase in salt concentration above 1 M.

The experiments described above confirm the hypothesis that BC of PMCs depends on the amount of unbound PAH in the composition of microcapsules, since with an increase in the ionic strength of the solution, the number of such sites of the polyelectrolyte became larger. These data could be used to preserve the properties of encapsulated substances during their encapsulation and incubation in solutions of various ionic strengths [34,35].

### 2.3. Study of Buffer Capacity of PMC Temperature Treatment

**Figure 3:**
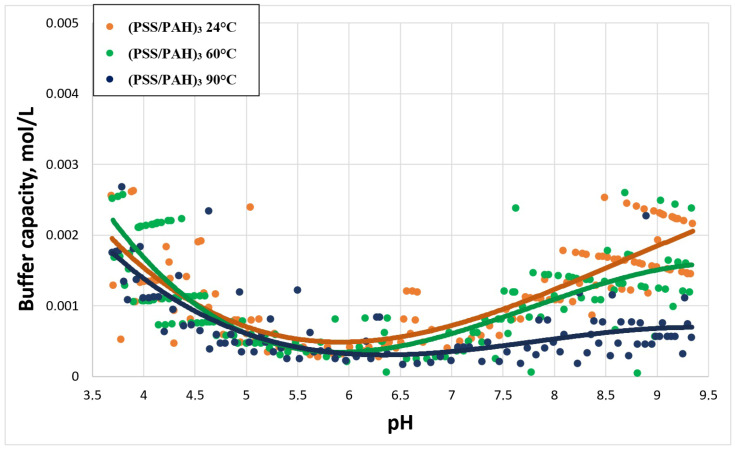
Buffer capacity of PMC composition (PSS/PAH)_3_ at different pH after hour incubation at the 24 °C, 60 °C and 90 °C.

The next stage of the study was the study of BC of PMCs after 60-min incubation at temperatures of 60 and 90 °C. As shown in [23], when heated, polyelectrolyte microcapsules shrink, and their shell thickens and compacts. At temperatures from 24 to 60 °C, the diameter of polyelectrolyte capsules and the thickness of their shell differ. From 60 to 90 °C, changes in values were observed with micro-differential scanning calorimetry [26]. In this regard, heating of PMCs could be considered as the opposite process to the addition of salt to the capsule suspension, which reduced the amount of unbound PAH in the capsules. Figure 3 shows the curves of the dependence of the buffer capacity of the PMC composition (PSS/PAH)_3_ on pH at different temperatures of preheating.

As can be seen from the figure, the buffer capacity in the alkaline pH range decreased with an increase in the heating temperature of the microcapsules. This was due to the fact that, at higher heating temperatures, PMCs were more strongly compressed and compacted, and, as a result, the amount of free PAH decreased.

**Figure 4:**
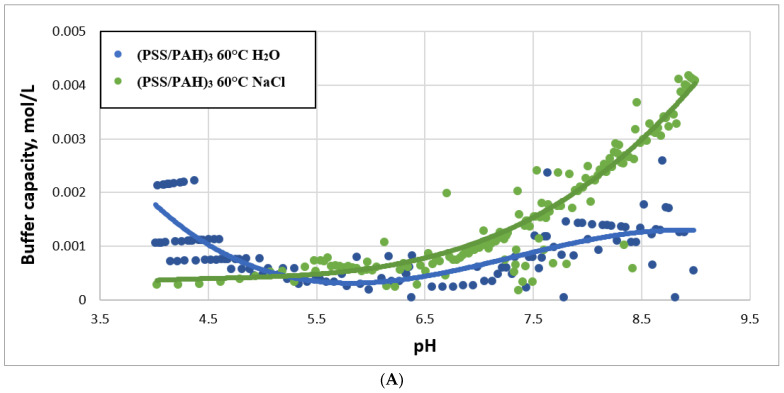

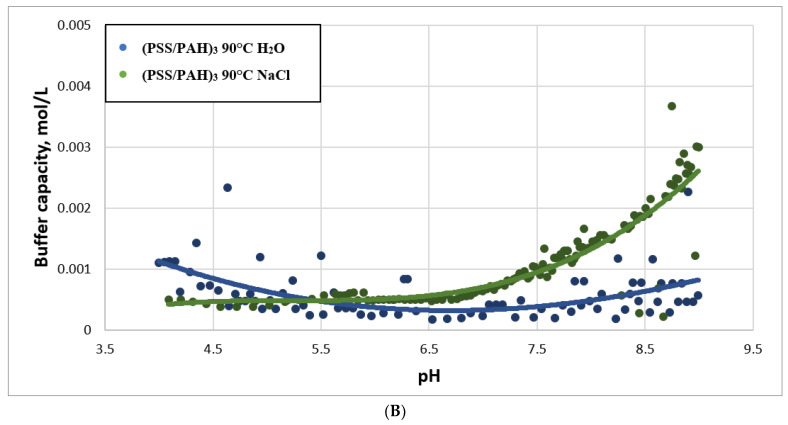
Buffer capacity of PMC composition (PSS/PAH)_3_ in water and in 1 M NaCl solution after incubation at 60 °C (**A**) and 90 °C (**B**).

Next, we studied BC of PMCs pre-heated at 60 °C and 90 °C, followed by the addition of 1 M NaCl. The results of this study are shown in Figure 4.

As can be seen from the figure, the addition of salt to preheated microcapsules led to an increase of BC, and an increase in the ionic strength of the solution led to the destruction of salt bridges between PAH and PSS, which were compensated by heating.

The obtained results may allow the adapting of the encapsulation and decapsulation methods of substances based on heating of PMCs. It would enable the saving of the functional properties of encapsulated molecules, for example, in self-healing materials and drug delivery systems after heating [36,37,38].

## 3. Materials and Methods

### 3.1. Materials

Polyelectrolytes polystyrenesulfonate sodium (PSS) and polyallylamine hydrochloride (PAH) with a molecular mass of 70 kDa, ethylenediaminetetraacetic acid disodium salt dihydrate (EDTA) were purchased from Sigma (St. Louis, MO, USA), and sodium chloride, sodium sulfate, sodium carbonate, and calcium chloride from “Reahim”.

### 3.2. Preparation of CaCO_3_ Microspherulites

At stirring of 0.33 M Na_2_CO_3_ the 0.33 M CaCl_2_ was added. The stirring time was 30 s. The suspension was maintained until complete precipitation of the formed particles. The process of “ripening” of microspherulites was controlled with the help of a light microscope. Then, the supernatant was decanted, the precipitate was washed with water and used to prepare PMC. The microparticles were obtained with an average diameter of 4.5 ± 1 μm.

### 3.3. Preparation of Polyelectrolyte Microcapsules

The polyelectrolyte microcapsules were obtained by layer-by-layer adsorbing the negatively or positively charged polyelectrolytes onto CaCO_3_ microspherulites, followed by dissolution of CaCO_3_. At the moment of dissolution of the CaCO_3_ core the inner space of the PMC was filled by inter-polyelectrolyte complex. Layer-by-layer adsorption of PAH and PSS on the CaCO_3_ microspherulites surface was carried out in polyelectrolyte solutions (concentration 2 mg/mL + 0.5 M NaCl). After each adsorption the CaCO_3_ particles with adsorbed polyelectrolytes were triple washed with a 0.5 M NaCl solution, which was necessary to remove un-adsorbed polymer molecules. The particles were separated from the supernatant by centrifugation. After applying the required number of layers, the carbonate kernels were dissolved in a 0.2 M EDTA solution for 12 h. The resulting capsules were washed three times with water to remove core decay products. The microcapsules were obtained with an average diameter of 4.5 ± 1 μm. The size and number of microcapsules was measured using the dynamic light scattering method on a Zetasizer nano ZS device (Malvern, UK).

### 3.4. Temperature Treatment

For heating, the microcapsule suspensions were incubated at the respective temperature in a SONIRET thermostat for a 60 min periud.

### 3.5. Measurement of Buffering Capacity

A suspension of microcapsules (PSS/PAH)_3_ (6.6 × 10^9^ microcapsules in 8 mL of water) was titrated with acid or base solutions in the pH range 4 to 9. Titration was done by manual measurement using a pH meter model Hanna pH 211. The acid or alkali (with a concentration of 0.001 M or 0.005 M) was added to the solution to change the pH of a solution by 0.02 or more. Buffering capacity was calculated from Equation (1), after estimating the slope of the titration curves at each point by the variation of the pH between previous and subsequent injections [39]:

(1) $$BC=\frac{C_{acid\;or\;alkali}V_{acid\;or\;alkali}\;}{V_{s}\left(pH\left(i+1\right)-pH\left(i-1\right)\right)}$$

*BC*—buffer capacity

*C_acid or alkali_*—concentration of HCl of NaOH

*V_acid or alkali_*—volume of HCl of NaOH

*V_s_*—volume of solution

*i*—number of titrations

## 4. Conclusions

In the course of this work, we studied the BC of PMCs’ composition (PSS/PAH)_3_ depending on the concentration, and type of salt, as well as preheating of the PMCs. It was found that with an increase in the concentration of NaCl to 1 M the BC of PMCs grew at pH ≥ 5, and an increase in salt concentration above 1 M did not change the BC. Comparison of BC microcapsules in the presence of mono- and di-valent salts of the same ionic strength showed that there was no noticeable difference in almost the entire pH range studied. Only at pH below 4.7 were differences observed that were directly related to BC and the saline solutions themselves. Researching the BC of preheated PMCs showed that it fell more strongly the higher the heating temperature was. The addition of 1 M sodium chloride to heated PMCs led to an increase in the BC. The effects described above confirmed the hypothesis we put forward in our previous work that the buffer properties of microcapsules are determined by uncompensated PAH regions in their composition.

The obtained results are necessary regarding effective encapsulation of pH-sensitive compounds, both in terms of saving functional properties of the encapsulated molecules and for creating a buffer barrier (metals, enzymes, polyelectrolytes, drugs). Furthermore, our results will allow the behavior of PMC and encapsulated substances, depending on the protonation/deprotonation level in different conditions of ionic strength and temperature, to be taken into account.

## Data Availability

Not applicable.
